# Acute *β*-*N*-Methylamino-L-alanine Toxicity in a Mouse Model

**DOI:** 10.1155/2015/739746

**Published:** 2015-10-29

**Authors:** Maitham Ahmed Al-Sammak, Douglas G. Rogers, Kyle D. Hoagland

**Affiliations:** ^1^Tropical Biological Disease Researches Unit, College of Science, University of Baghdad, Baghdad, Iraq; ^2^School of Natural Resources, University of Nebraska-Lincoln, Lincoln, NE 68583, USA; ^3^School of Veterinary Medicine and Biomedical Sciences, University of Nebraska-Lincoln, Lincoln, NE 68583-0905, USA

## Abstract

The cyanobacterial neurotoxin *β*-*N*-methylamino-L-alanine (BMAA) is considered to be an “excitotoxin,” and its suggested mechanism of action is killing neurons. Long-term exposure to L-BMAA is believed to lead to neurodegenerative diseases including Parkinson's and Alzheimer's diseases and amyotrophic lateral sclerosis (Lou Gehrig's disease). Objectives of this study were to determine the presumptive median lethal dose (LD_50_), the Lowest-Observed-Adverse-Effect Level (LOAEL), and histopathologic lesions caused by the naturally occurring BMAA isomer, L-BMAA, in mice. Seventy NIH Swiss Outbred mice (35 male and 35 female) were used. Treatment group mice were injected intraperitoneally with 0.03, 0.3, 1, 2, and 3 mg/g body weight L-BMAA, respectively, and control mice were sham-injected. The presumptive LD_50_ of L-BMAA was 3 mg/g BW and the LOAEL was 2 mg/g BW. There were no histopathologic lesions in brain, liver, heart, kidney, lung, or spleen in any of the mice during the 14-day study. L-BMAA was detected in brains and livers in all of treated mice but not in control mice. Males injected with 0.03 mg/g BW, 0.3 mg/g BW, and 3.0 mg/g BW L-BMAA showed consistently higher concentrations (*P* < 0.01) in brain and liver samples as compared to females in those respective groups.

## 1. Introduction

**Figure 1:**
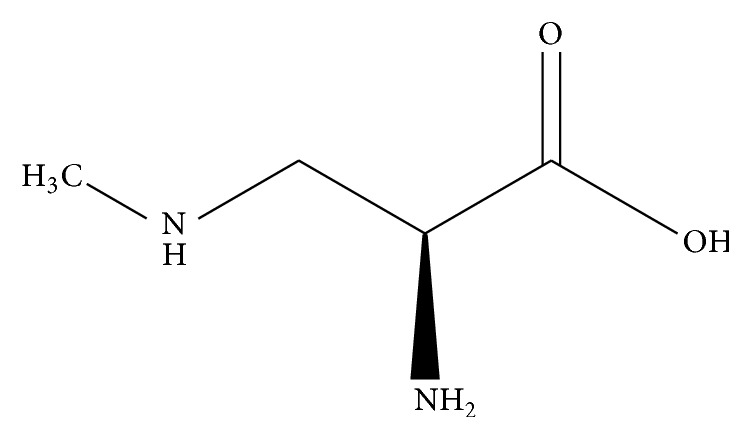
L-BMAA chemical structure.

*β*-N-Methylamino-L-alanine (BMAA) is a nonprotein amino acid. Its molecular formula is C_4_H_10_N_2_O_2_ (Figure 1), its CAS number is 15920-93-1, and it has a molar mass of 118.13 g/mol. The naturally occurring isomer of BMAA is L-BMAA [1]. Exposure to L-BMAA may cause neuronal death in the brains of humans and animals, and exposure may play a role in the pathogenesis of Parkinson's and Alzheimer's diseases and amyotrophic lateral sclerosis or Lou Gehrig's disease.

Various doses of L-BMAA have been administered intraperitoneally (IP) to mice, rats, and chickens [1–3], orally to monkeys and mice [4–6], and intracerebroventricularly to mice and rats [7–11]. Several studies demonstrated that a single dose of L-BMAA causes hyperexcitability, inability to extend the legs, a dragging gait, myoclonus, and convulsions [1, 2, 7].

Additional studies demonstrated similar findings, and this is likely because L-BMAA crosses the blood-brain barrier and causes irreversible damage [3, 8, 12, 13]. In rats, L-BMAA adversely affects monoamine neurons in the substantia nigra, and it decreases noradrenaline levels in the hypothalamus [13].

Clinical symptoms caused by L-BMAA neurotoxicity may be due to changes in cholinergic and glutamatergic neurotransmission, primarily due to a decrease in the number of glutamatergic receptors [9–11]. Glutamate acts on the ligand-gated receptor channels N-methyl-D-aspartic acid (NMDA), 2-amino-3-(5-methyl-3-oxo-1,2-oxazol-4-yl) propionic acid (AMPA), and kainate receptors at the postsynaptic membrane. This is an important mechanism in memory function and for the sensing of environmental cues [14–18]. Because L-BMAA has glutamate receptor agonist activity in mammals and plants [19, 20], Couratier et al. [21] suggested that neuronal degeneration in ALS is initiated at the level of glutamate AMPA/kainate receptors. The initiation of neurotoxicity may be due to direct action on NMDA receptors and activation of metabotropic glutamate receptors 5 (mGluR5) and/or by inducing oxidative stress [22–26].

Additional studies demonstrated that L-BMAA inhibits the cysteine/glutamate antiporter (system Xc-) that mediates cysteine uptake and increases oxidative stress [26], and/or it may activate AMPA/kainate receptors which causes selective death of motor neurons [24]. L-BMAA concentrations as low as 30 *μ*M cause motor neuron death [24], a concentration of 10 *μ*M enhances neuronal death [25, 26], and a concentration of 3 mM causes death of entire cortical neuron populations [27]. L-BMAA is actively transferred across the blood-brain barrier [28] where it also increases output of dopamine by affected neurons [29].

Fruit bats, a known source of L-BMAA, are used to make a traditional soup by the Chamorro people in Guam [30, 31]. Cox and Sacks [32] calculated that a human weighing 70 kg body weight (BW) and eating two fruit bats of 500 g each will ingest approximately 28 mg L-BMAA/kg BW [32]. Perry et al. [5], however, did not observe any behavioral, neurochemical, or neuropathological changes in mice orally dosed with 0.5 mg of L-BMAA/g BW/day for 11 weeks, and lesions were not seen histopathologically in the brain. Cruz-Aguado et al. [6] also did not observe neuronal damage after orally dosing mice with 1 mg LBMAA (28 mg/kg daily) for 30 days.

Spencer et al. [4] were the first to demonstrate that administering 100–315 mg L-BMAA/kg daily for up to 12 weeks to monkeys caused clinical symptoms very similar to amyotrophic lateral sclerosis/Parkinson dementia complex (ALS/PDC), and, in another study [4], they suggested a significant role for L-BMAA in the etiology of ALS-PDC [4]. However, Dawson Jr. et al. [33] reported sex-dependent changes in motor function and spinal cord neurochemistry, and they concluded that these changes were not related to ALS/PDC. More recently, L-BMAA has been detected in many fish species and aquatic plants, as well as open water samples from reservoirs in Nebraska, USA, which suggests that a direct human exposure to L-BMAA may occur through the food chain [33, 34].

Although earlier studies have investigated the acute neurotoxic effects of L-BMAA in several animal species [35], Buenz and Howe [36] demonstrated that direct administration of BMAA to the mouse's brain causes sporadic death of hippocampal neurons, and they demonstrated this histopathologically using TUNEL staining after subcutaneous injection of 600 mg/kg of L-BMAA in rat pups.

The objectives of the present study were to determine the presumptive LD_50_ and LOAEL for L-BMAA in mice. In addition, brain and other selected tissue specimens were examined for histopathologic lesions.

## 2. Materials and Methods

### 2.1. L-BMAA and Doses

Five doses of L-BMAA (supplied by the Institute for Ethnomedicine, Jackson Hole, WY, USA) were prepared in sterile water and adjusted to pH 6.5 with NaOH [1] prior to IP injection.

Each dose was dissolved in 0.5 mL of sterile H_2_O and calculated as follows depending on each mouse weight:

 Dose (mg/g BW) × mouse weight (15–20 g) = *Y* mg/mouse (L-BMAA/mouse), for example, Group E Dose (3 mg/g BW) × 18 g = 54 mg/0.5 mL.

### 2.2. Animals and Housing

Thirty-five male and 35 female NIH Swiss Outbred mice (Harlan Laboratories, Indianapolis, IN, USA) weighing 15–20 g each were used. Males and females were housed separately, and they were fed a standard mouse diet and water ad libitum. The mice were allowed to acclimate for 3 days prior to IP injection, and they were housed and treated according to University of Nebraska-Lincoln Institutional Animal Care and Use Committee guidelines.

### 2.3. Study Design

Duration of the study was 14 days. Mice were randomly assigned to six groups, and equal numbers of males and females were included in each group (Table 1). Each mouse was injected with 0.5 mL of the respective L-BMAA preparation depending on individual body weight and observed 4 times daily for 14 days; control mice received sterile water only.

### 2.4. Presumptive Median Lethal Dose (LD_50_) and the Lowest-Observed-Adverse-Effect Level (LOAEL)

Presumptive LD_50_ was defined as the L-BMAA dose that caused one-half of the mice receiving that dose to display clinical symptoms requiring humane euthanasia. The LOAEL was defined as the lowest dose of L-BMAA that caused adverse clinical symptoms after IP injection [37].

### 2.5. Tissue Samples and Histopathology

Mice displaying adverse clinical symptoms were humanely euthanized, and all remaining mice were euthanized 14 days after injection. Specimens of brain, liver, lung, heart, spleen, and kidney from one male and one female in each group were immediately immersed in 10% phosphate-buffered formalin. After 24 hours of fixation, brains were transversely sectioned at five levels in a cranial to caudal fashion, and then all specimens were routinely processed for histopathology. Sections of each specimen were cut at 4-5 *μ*m and stained with hematoxylin and eosin. Brain and liver specimens from all remaining mice were frozen for L-BMAA extraction [3].

### 2.6. L-BMAA Extraction

Frozen liver and brain specimens from 20 mice representing Control, A, B, and E group (Table 2) were thawed, and 50 mg of the frontal brain lobe and 50 mg of the liver from each mouse were weighed separately in 2 mL glass amber tubes.

Five hundred *μ*L of 6 M HCl was added, and the mixture was sonicated for 30 seconds and then vortexed. After heating for 16 hours at 110°C, 100 *μ*L of the mixture was transferred to a Millipore Ultrafilter centrifuge tube (EMD Millipore Corporation, Billerica, MA, USA) and centrifuged for 3 min at 1300 rpm. The filtered solution was then evaporated to dryness by using N_2_ gas and heating at 45°C [34].

### 2.7. L-BMAA Detection Methods

Brain and liver samples from the 20 male and female mice (Table 2) were dried using established protocol and analyzed using Ultra Performance Liquid Chromatography (UPLC), and Liquid Chromatography/Mass Spectrometry/Mass Spectrometry (LC/MS/MS) at the Institute for Ethnomedicine (IEM), Jackson Hole, WY, USA [38, 39].

#### 2.7.1. Ultra Performance Liquid Chromatography Method (UPLC-UV)

L-BMAA was detected in mouse tissues using Waters Acquity pressure UPLC-UV and in-line single quadrupole mass spectrometry (UPLC-MS) following a validated method [39]. Hydrolyzed samples were derivatized with AQC. Eluents were purchased from Waters (Eluent A: part #186003838, Eluent B: part #186003839); composition is proprietary. Separation was achieved by reverse phase chromatography over 9.5 minutes on a Waters AccQ-Tag Ultra column (part #186003837, 2.1 × 100 mm) at temperature of 55°C and flow rate of 0.7 mL/min. The elution gradients were 0.0 min = 0.1% B; 0.54 min = 0.1% B curve 6; 6.24 min = 9.1% B curve 7; 7.74 min = 21.2% B curve 6; 8.04 min = 59.6% B curve 6; 8.73 min= 0.1% B curve 6; 9.5 min = 0.1% B curve 6. L-BMAA was identified using mass and retention time in comparison with an authenticated L-BMAA standard [38, 40–42].

#### 2.7.2. Liquid Chromatography-Tandem Mass Spectrometry Method (LC/MS/MS)

Hydrolyzed samples were derivatized with 6-aminoquinolyl-N-hydroxysuccinimidyl carbamate (AQC). L-BMAA analyses were performed using a triple quadrupole instrument (Thermo Scientific Finnegan TSQ Quantum UltraAM, San Jose, CA) and separation was performed by using Waters Acquity-UPLC system with a Binary Solvent Manager, Sample Manager, and a Waters AccQ-Tag Ultra column (part #186003837, 2.1 × 100 mm) at 55°C. Separation was completed using 0.65 mL/min in aqueous 0.1% (v/v) formic acid (Eluent A) and 0.1% (v/v) formic acid in acetonitrile (Eluent B) with the following elution gradient: 0.0 min = 99.1% A; 0.5 min = 99.1% A curve 6; 2 min = 95% A curve 6; 3 min = 95% A curve 6; 5.5 min = 90% A curve 8; 6 min = 15% A curve 6; 6.5 min = 15% A curve 6; 6.6 min = 99.1% A curve 6; 8 min = 99.1% A curve 6. The heated electrospray ionization (H-ESI) probe was supplied with nitrogen gas at a nebulizing pressure of 40 psi and a vaporizer temperature of 400°C.

The mass spectrometer operating conditions were as follows: capillary offset of 35, capillary temperature of 270°C, auxiliary gas pressure of 35, spray voltage 3500 V, source collision energy of 0 eV, multiplier voltage of −1719 V, and tube lens offset of 110. The second quadrupole was pressurized to 1.0 m Torr with argon. In the first quadrupole filter, ion* m/z* 459 was isolated as the precursor ion and subjected to collision induced dissociation (CID). The second step mass filtering was completed using selective reaction monitoring (SRM) of L-BMAA after CID in the collision cell.

The* m/z* transitions monitored were 459–119 CE 21 eV; 459–289 CE 17 eV; 459–171 CE 38 eV. The three product ion resultants originating from derivatized L-BMAA (119, 289, and 171* m/z*) were scanned and detected by the third quadrupole and their relative abundances quantified. The ratio of the three product ions was compared to the product ion ratios of an authenticated L-BMAA standard [38–41].

### 2.8. Statistical Analysis

Results were presented as mean ± standard deviation (SD) using three results' reading. The statistical analysis was used in our study to show the degree of significance of our data.

## 3. Results

### 3.1. Presumptive Median Lethal Dose (LD_50_)

The presumptive LD_50_, the dose at which 50% of the mice became moribund and required euthanasia, was 3 mg/g L-BMAA BW. Four males and three females in the group given this dose developed myoclonus, convulsions, and uncontrolled urination and defecation within 20 minutes of injection.

### 3.2. Lowest-Observed-Adverse-Effect Level (LOAEL)

The lowest administered dose that caused the previously described clinical symptoms was 2 mg/g L-BMAA BW; one female mouse in the group given this dose required euthanasia 45 minutes after injection.

### 3.3. Gross Examination and Histopathology

There were no gross lesions at necropsy in any of the mice. There were no histopathologic lesions in the brain, liver, lung, kidney, heart, or spleen from any of the mice, including one male and one female in the 3 mg L-BMAA/g BW group and the one female in the 2 mg L-BMAA/g BW group that had displayed adverse clinical symptoms.

### 3.4. L-BMAA in Brain and Liver Specimens

#### 3.4.1. Ultra Performance Liquid Chromatography Results

**Figure 2:**
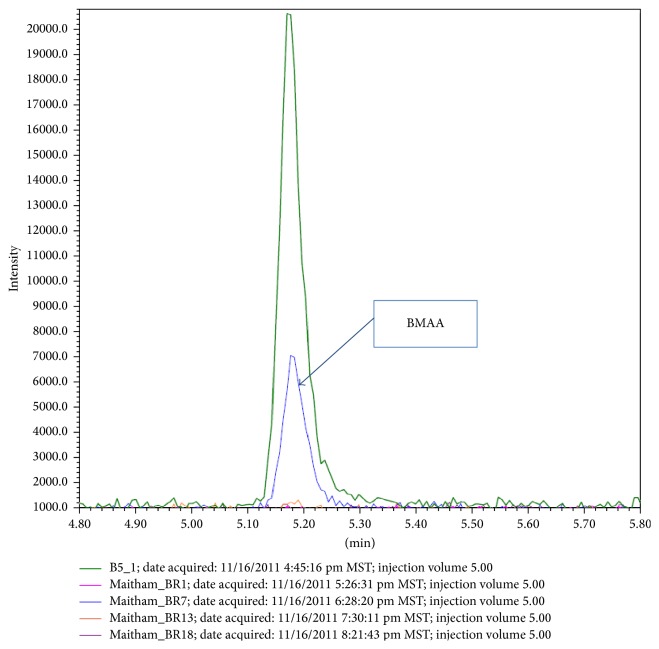
UPLC chromatography of L-BMAA level (4.66 *μ*g/g) in brain of male mouse with dose 3 mg/g BW.

**Figure 3:**
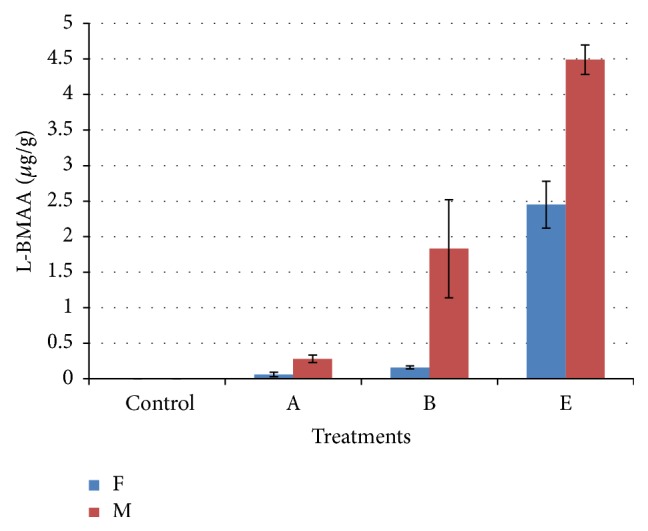
Comparison of L-BMAA concentrations (*μ*g/g) in brains of male and female mice as detected by UPLC-UV. T-bars represent the mean of *n*  (*n* = 3)   ± SD. A, B, and E: mice treated (0.03, 0.3, and 3 mg/g BW) groups.

**Figure 4:**
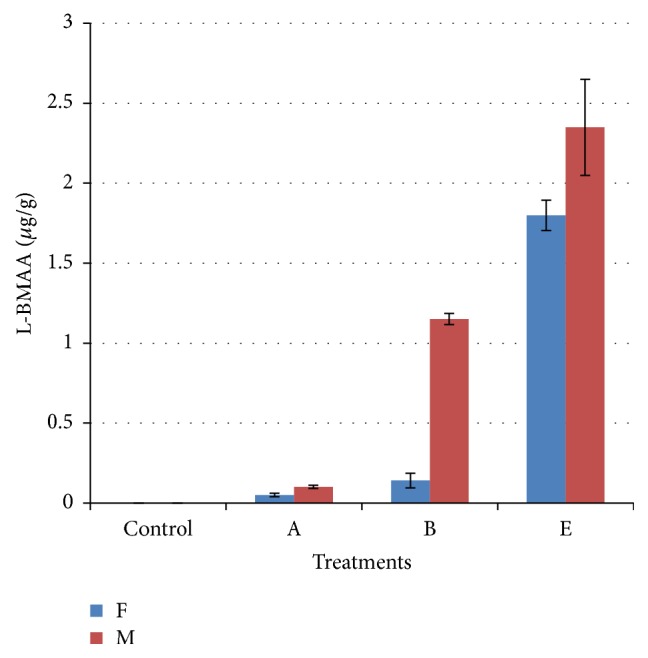
Comparison of L-BMAA concentrations (*μ*g/g) in livers of male and female mice as detected by UPLC-UV. T-bars represent the mean of *n*  (*n* = 3) ± SD. A, B, and E: mice treated (0.03, 0.3, and 3 mg/g BW) groups.

Table 3 shows L-BMAA concentrations in brains and livers as detected by UPLC-UV, with the concentrations of L-BMAA in male subgroups being higher than that in female subgroups.  Figure 2 will show UPLC-UV chromatographic detection figure of L-BMAA in male mouse brain injected with 3 mg/g BW. Figures 3 and 4 will show UPLC-UV results comparison of L-BMAA concentrations in (*μ*g/g) in brains and liver of male and female mice.

#### 3.4.2. Liquid Chromatography-Tandem Mass Spectrometry Results

**Figure 5:**
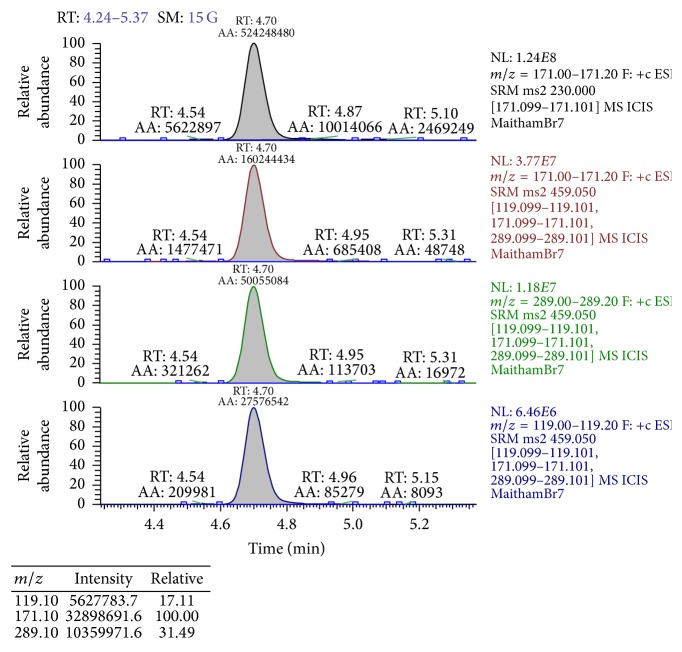
LC/MS/MS chromatography of L-BMAA level (33.6 *μ*g/g) in male mouse brain tissue with a dose of 3 mg/g BW.

**Figure 6:**
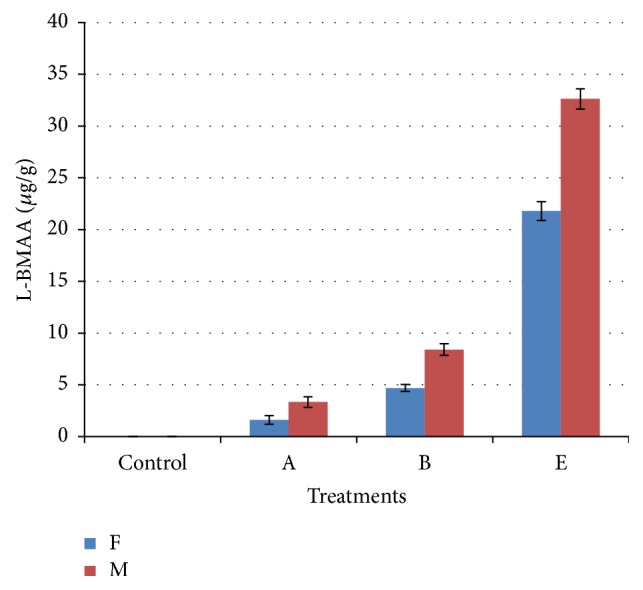
Comparison of L-BMAA concentrations (*μ*g/g) in brains of male and female mice as detected by LC/MS/MS chromatography. T-bars represent the mean of *n*  (*n* = 3) ± SD. A, B, and E: mice treated (0.03, 0.3, and 3 mg/g BW) groups.

**Figure 7:**
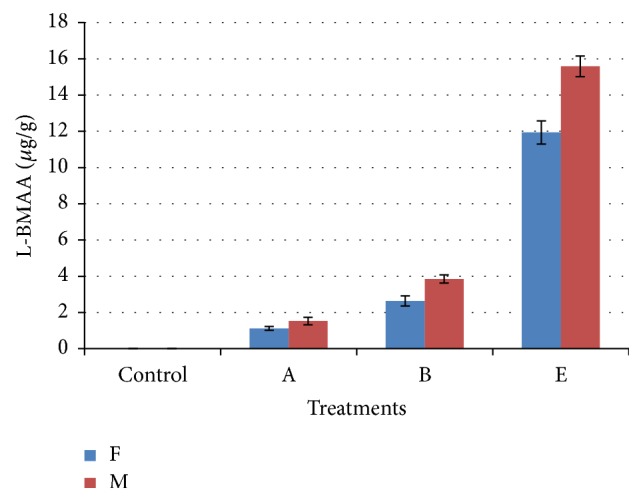
Comparison of L-BMAA concentrations (*μ*g/g) in livers of male and female mice as detected by LC/MS/MS chromatography. T-bars represent the mean of *n*  (*n* = 3) ± SD. A, B, and E: mice treated (0.03, 0.3, and 3 mg/g BW) groups.

To confirm the results of L-BMAA concentrations detected in extracted brain and liver samples from treated and control mice using UPLC-UV, LC/MS/MS chromatography was performed on these same samples. Results are given in Table 4.  Figure 5 will show LC/MS/MS chromatographic detection figure of L-BMAA in male mouse brain injected with 3 mg/g BW. Comparison of L-BMAA concentrations in male and female brains and livers using LC/MS/MS chromatography is shown in Figures 6 and 7, respectively.

## 4. Discussion

Results of this study indicate that the presumptive LD_50_ of L-BMAA is 3 mg/g BW and the LOAEL of L-BMAA is 2 mg/g BW in male and female NIH Swiss Outbred mice when administered intraperitoneally. Clinical symptoms such as myoclonus, convulsions, and uncontrollable urination and defecation seen in some of the mice are symptoms similar to those reported by other investigators [2, 7, 8] regardless of animal species used or routes of L-BMAA administration [43]. Other symptoms included a dragging gait, weakness, and convulsions [2]; hyperexcitability and shaking [7]; ataxia, rolling, unsteady gait, and myoclonia [8]. Studies that did not report any clinical symptoms or neuropathological changes presumably used insufficient doses of BMAA or routes of administration, that is, mainly by mouth [5, 6, 43]. Tissue sections were cut at 4-5 *μ*m and stained with hematoxylin and eosin. Only the front lope of brain and a part of the liver were used to measure the accumulated BMAA from our original doses [44]. This lack of histopathologic lesions in the brain may not be uncommon. However, Pablo et al. [44] failed to detect lesions in brain samples taken from ALS and AD deceased patients. Also, Perry et al. [5] could not detect any neurochemical and neuropathological changes in BMAA orally exposed mice [6]. In addition, no histopathologic changes were seen in the hippocampus of neonatal rats injected subcutaneously with 0.2 mg/g BW BMAA [45]. Exposure to BMAA is believed to kill neurons in the brain, leading to neurodegenerative diseases. In one study, neuronal death occurred within 24 hours after 0.6 mg/g BW BMAA was injected subcutaneously in neonatal rats.

The results showed a significantly higher concentration of L-BMAA, again demonstrating that the LC/MS/MS was more sensitive than the UPLC-UV method. Other investigators have reported that IP administration of L-BMAA causes selective degeneration of cerebellar cortical neurons in rat pups [3] and intracerebral administration of L-BMAA causes sporadic death of hippocampal neurons in mice [36].

Histopathologic lesions were not seen in brains or in other selected tissues from treated mice, including symptomatic mice, in the present study. This lack of histopathologic lesions might suggest that L-BMAA causes a “biochemical lesion” as opposed to a histopathologic lesion in brain and other tissues under the conditions of this study. To truly focus on possible histopathologic changes caused by L-BMAA in tissues from mice, however, future studies might examine larger numbers of mice and employ perfusion fixation and advanced staining techniques. Buenz and Howe [36] demonstrated that direct administration of L-BMAA to the mouse's brain causes sporadic death of hippocampal neurons, and they demonstrated this histopathologically using TUNEL staining after subcutaneous injection of 600 mg/kg of L-BMAA in rat pups [45]. The method of administrating L-BMAA was intraperitoneal injection which was found to be accurate to induce acute neurotoxicity in mice brain as was shown in a study in 1990 [3].

LC/MS/MS chromatography was shown to be more sensitive (*P* < 0.01) than UPLC-UV when determining L-BMAA concentrations in tissues. The use of both methods consistently detected higher concentrations of L-BMAA in brain and liver samples from males in Groups A (0.03 mg/g BW), B (0.3 mg/g BW), and E (3.0 mg/g BW) as compared to females in those groups (*P* < 0.01) which could be result of lower metabolic rate in female than in male [46, 47].

## 5. Conclusions

In conclusion, the presumptive LD_50_ of L-BMAA is 3 mg/gm BW and the LOAEL of L-BMAA is 2 mg/g BW in male and female NIH Swiss Outbred mice when administered intraperitoneally, and this animal model may prove useful for investigating the role of L-BMAA in neurodegenerative diseases. LC/MS/MS chromatography is more sensitive (*P* < 0.01) than UPLC-UV when determining L-BMAA concentrations in mouse tissues.

Future studies should further examine whether L-BMAA concentrations in brain, liver, and perhaps other tissues are influenced by gender. Studies might be designed to examine the long-term, chronic effects of administering L-BMAA at doses lower than the LOAEL for longer than 14 days. Perhaps then a relationship between L-BMAA exposure and neurodegenerative diseases in humans can be determined. L-BMAA has been shown clearly to be a neurotoxin in our mouse model and in other laboratory animals. This reinforces the notion that L-BMAA can cause serious neurodegenerative diseases in humans (and animals) as it can pass easily through the blood-brain barrier [12], and it can also transfer from mother to offspring through their milk [48]. When administered intraperitoneally to mice at doses greater than 2 mg/g BW, the onset of adverse clinical symptoms is rapid. A long-term study of the relation between human neurodegenerative diseases such as ALS and the presence of BMAA in the same geographic environment can help us to determine the effect of the cyanobacterial neurotoxin (BMAA) on human in USA and worldwide, similar to the one recently done by Delzor and his colleagues in France [49].

## Figures and Tables

**Table 1:** L-BMAA doses and study design. BW: body weight.

Group	L-BMAA dose (mg/g BW)	Number of mice	
		Male	Female
A	0.03	7	7
B	0.3	7	7
C	1	6	6
D	2	6	6
E	3	6	6
Control	0	3	3

**Table 2:** Number of brain and liver specimens collected from each mice group.

Group	L-BMAA dose mg/g BW	Female	Male
Control	0	1	1
A	0.03	3	3
B	0.3	3	3
E	3	3	3

**Table 3:** L-BMAA concentrations (*μ*g/g) in brains and livers of mice as detected by UPLC-UV. Each value is the mean of three replicates ± standard deviation. BW: body weight; F: female; M: male. A, B, and E: mice treated (0.03, 0.3, and 3 mg/g BW) groups.

L-BMAA dose (mg/g BW)	Gender	L-BMAA (*µ*g/g) in brain	L-BMAA (*µ*g/g) in liver
Control (0)	F	0.00 ± 0.00	0.00 ± 0.00
	M	0.00 ± 0.00	0.00 ± 0.00

(A) 0.03	F	0.06 ± 0.03	0.05 ± 0.01
	M	0.28 ± 0.05	0.10 ± 0.01

(B) 0.30	F	0.16 ± 0.02	0.14 ± 0.05
	M	1.83 ± 0.69	1.15 ± 0.04

(E) 3.00	F	2.45 ± 0.33	1.80 ± 0.09
	M	4.49 ± 0.21	2.35 ± 0.30

**Table 4:** L-BMAA concentrations (*μ*g/g) in brains and livers of mice as detected by LC/MS/MS chromatography. Each value is the mean of three replicates ± standard deviation. BW: body weight; F: female; M: male. A, B, and E: mice treated (0.03, 0.3, and 3 mg/g BW) groups.

L-BMAA dose (mg/g BW)	Gender	L-BMAA (*µ*g/g) in brain	L-BMAA (*µ*g/g) in liver
Control (0)	F	0.00 ± 0.00	0.00 ± 0.00
	M	0.00 ± 0.00	0.00 ± 0.00

(A) 0.03	F	1.61 ± 0.41	1.12 ± 0.10
	M	3.34 ± 0.52	1.53 ± 0.21

(B) 0.30	F	4.69 ± 0.34	2.64 ± 0.28
	M	8.41 ± 0.56	3.85 ± 0.22

(E) 3.00	F	21.79 ± 0.92	11.94 ± 0.64
	M	32.62 ± 0.99	15.59 ± 0.57
